# Treatment of Epithelioma by Radium

**Published:** 1917-05

**Authors:** Russell H. Boggs


### Treatment of Epithelioma by Radium.

BY RUSSELL H. BOGGS.

The writer emphasizes the fact in the *International Clinics* with many photographic illustrations that in each case the proper form of radiation and dosage for each case must be carefully determined.

Four classes of epithelioma are to be considered:

1. The lesion which can be cured by one application of radium with the proper dosage.

2. The lesion which is so situated that glandular involvement is likely to take place or has already occurred and the Roentgen ray should be employed as an adjunct to treat adjacent glands.

3. Those cases in which the local application of radium supplemented by the Roentgen ray will only act as a palliative measure.

4. Those cases in which excision is justified to be followed by radiotherapy.

Professor Boggs believes that radium and the X-ray should always be considered first in the treatment of epithelioma, because, when properly applied, practically all epitheliomatous tissue can be made to disappear and there are fewer recurrences than by any other method. In order to apply the method, however, the operator must have the required clinical experience with these growths as well as a knowledge of the use of the agents employed.

Inoperable cases in which the tonsil is involved are often markedly improved so far as symptoms are considered.—*International Clinics.*

---

The eighteenth annual meeting of the American Proctologic Society will be held at Detroit, Mich., June 12 and 13, 1916.

